# Social capital and its influence on sustainable economic development and natural resource management among ethnic minorities in Phu Tho province, Vietnam

**DOI:** 10.1371/journal.pone.0347481

**Published:** 2026-04-24

**Authors:** Van Khoat Vu, Kim Hanh Vu, Thi Thu Nga Vu

**Affiliations:** Faculty of Transport Safety and Environment, University of Transport and Communications, Hanoi, Vietnam; Center for Research and Technology Transfer, VIET NAM

## Abstract

This study investigates the role of social capital in promoting sustainable economic development and natural resource management among ethnic minority communities in Tan Son and Thanh Son districts, Phu Tho province, Vietnam. Located near Xuan Son National Park, these communities depend on natural resources and are increasingly involved in ecotourism. Using a mixed-methods approach, data were collected from 355 households through surveys, interviews, and focus groups. Results show high levels of mutual trust (80.6%) and close interpersonal relationships (71%), which facilitate collective action and information sharing. Participation in social organizations – particularly the Farmers’ and Women’s Unions – plays a crucial role in mobilizing community efforts and enhancing environmental awareness. However, information exchange remains mostly within family and ethnic lines, reflecting limited bridging capital. A linear regression model confirms that trust, social ties, and organizational involvement are significantly associated with household participation in forest management. While bonding capital is positively related to internal cohesion, weak external linkages may hinder broader collaboration and innovation. The study found that enhancing social capital, especially bridging and linking forms, is statistically linked to advancements in inclusive and sustainable development in buffer zones of protected areas.

## 1. Introduction

Sustainable development has increasingly become central to Vietnam’s national policy as the country seeks to balance economic growth with long-term environmental protection and social well-being. The need to harmonize livelihood improvement with conservation of natural resources, particularly in rural and mountainous regions where communities still rely heavily on land, forests and biodiversity for their subsistence, has been emphasized in many policies of Vietnam, such as the National Action Plan for the Implementation of the 2030 Sustainable Development Agenda, the National Target Program for Sustainable Poverty Reduction in the 2021–2025 Period and the National Strategy for Biodiversity until 2030 and vision to 2050 [1–3]. Within this context, it is essential to understand the factors that enable communities to pursue the dual goals of development and conservation. Social capital is one of the factors shaping sustainable development outcomes in Vietnam. Social capital is broadly understood as “the norms, trust and networks that enable collective action for mutual benefit” [4]. It encompasses bonding ties within households and ethnic groups, bridging ties across occupational or community boundaries, and linking connections with institutions and external actors. Social capital is considered a form of resource that individuals utilize to achieve their livelihood goals. It is created through networks and interactions among individuals and social groups, characterized by trust-based relationships, mutual support, and exchanges grounded in shared values and social norms. Social capital serves as a critical resource in the implementation of economic, social, and environmental development programs [4,5].

Social capital is not only essential for economic and social development but also plays a critical role in the sustainable management of natural resources. In many rural and indigenous communities, especially those living in or near protected areas, social capital forms the foundation for collective action in the management of forests, water, land, and biodiversity. This is especially relevant in ecotourism contexts and in communities adjacent to national parks or nature reserves, where resource-based livelihoods intersect with conservation priorities [6–9]. Elinor Ostrom’s 1990 seminal work on the governance of common-pool resources highlighted the critical role of trust, norms, and social networks, the core elements of social capital, in enabling communities to manage natural resources sustainably without top-down regulation or privatization. Communities with strong bonding and bridging social capital are more likely to develop and enforce local rules, monitor use, and resolve conflicts effectively [10]. Social capital allows rural communities to cooperate in the management of water systems, forests, fisheries, and grazing lands. Mutual trust and shared social norms reduce transaction costs and promote long-term stewardship [7,11–13]. However, Recent studies also indicate that effective natural resource management depends not only on social dynamics but also on the integration of ecological factors into planning processes. Tampekis (2025) demonstrates that forest infrastructure, particularly road systems, should be designed with careful consideration of ecosystem vulnerability to reduce environmental impacts and support long-term sustainability. This perspective complements the role of social capital by underscoring the need for community-based management to be aligned with environmentally informed planning approaches [14]. In Vietnam and other Southeast Asian countries, village-level forest protection groups often function effectively when built on strong local relationships and shared responsibility, which are enhanced by social capital [7,15,16].

In ecotourism development, particularly in buffer zones of national parks, social capital plays a critical role in balancing conservation with livelihood generation. Communities with dense social networks and participatory institutions are better able to organize ecotourism services, share revenues fairly, and reinvest in both conservation and community development. Birendra and Kusi (2025) identified challenges such as resource constraints and human-wildlife conflicts and utilize visualization tools to map collaborative connections. The findings highlighted the importance of strengthening social capital within protected areas to enhance sustainable ecotourism practices [6]. A case study from the Tangkahan buffer zone of Leuser National Park in Indonesia illustrated how community-based ecotourism serves as a resource-sharing strategy. The research demonstrated that local communities, through strong social capital, can effectively manage ecotourism initiatives that contribute to both conservation efforts and community development [17]. Research by Jones (2005) in Namibia and by Stronza (2007) in the Amazon showed that ecotourism projects succeed when local people have strong internal cohesion and could build linkages with external actors, including NGOs, government agencies, and tourists. This enable the co-management of resources and enhances legitimacy and compliance with conservation goals [18,19]. In Vietnam, community-based tourism initiatives in regions like Sa Pa and Mai Chau have demonstrated how ethnic minority communities could leverage social capital – kinship ties, shared cultural values, and local leadership – to organize tourism services while conserving natural and cultural assets [20–22].

Strong social capital also improves environmental governance. Trust and cooperation facilitate inclusive decision-making, conflict resolution, and adaptive management. Bridging and linking capital enable marginalized groups to access information, voice concerns, and influence policy. The study examining the Wang Hilltop Pond Irrigation System in Guangdong, China, illustrated how strong social capital, rooted in lineage-based community ties, enabled residents to collaboratively revise water management rules in response to climate changes. This collective action, supported by trust and shared norms, led to the successful adaptation and sustained governance of the irrigation system [23]. In Brazil, research on a co-management approach in a state park revealed that building social capital through trust and equality among stakeholders improved rule enforcement and conflict resolution. The establishment of participatory institutions allowed for inclusive decision-making, demonstrating the effectiveness of social capital in adaptive co-management frameworks [24]. Research by Krishna (2002) in India found that higher social capital correlated with better local environmental management outcomes [25]. Similarly, Uphoff and Wijayaratna (2000) observed that in Sri Lanka, farmer groups with higher levels of internal trust were more effective in managing irrigation systems and sustaining agricultural productivity [26]. Additionally, social capital can help communities resist environmentally destructive activities, such as illegal logging or land grabbing, by fostering solidarity and collective resistance. In such contexts, community trust and shared values create a united front for advocacy and defense of territorial rights [27].

While social capital supports sustainability, it does not always produce positive outcomes. As noted earlier, excessive bonding capital can create closed groups that resist outside ideas, even if those ideas promote sustainability. If influential elders or community leaders, trusted figures in bonding capital, reject modern conservation practices or government policies, they may block progress [28]. This risk was particularly acute in ethnic minority communities where traditional authority structures were strong. Moreover, when bridging or linking social capital was weak, communities may face difficulties to access markets, policy support, or technical expertise needed for sustainable development. The unequal distribution of social capital can also reinforce existing power hierarchies, potentially excluding women, youth, or marginalized sub-groups from meaningful participation in decision-making processes [29].

Although some studies have qualitatively examined social capital in Vietnam’s ethnic minority regions, few have employed quantitative modelling to assess its association with household participation in forest and forestland management. This study addresses this gap by combining household survey data with regression analysis to quantify these relationships. This study addresses the following research question: How does social capital influence household participation in forest and land management among ethnic minority communities in Phu Tho Province, Vietnam?

This study is part of a broader research program examining institutional and socio-economic drivers of sustainable development in upland Vietnam. Although conducted in the same geographical region as a separate study by the authors, Vu et al., the present research constitutes a distinct investigation with a different theoretical foundation, unit of analysis, and research objective. The companion study examines institutional determinants of natural resource management effectiveness, focusing on governance performance and structural policy factors. In contrast, this manuscript is grounded in social capital theory and development economics, examining how relational assets – trust, reciprocity, community networks, and external linkages – shape household-level economic sustainability and environmental practices.

Importantly, the two studies do not examine the same conceptual constructs, or causal pathways. The companion paper evaluates institutional effectiveness at the resource management level, whereas this study analyzes sustainable economic outcomes and resource-use behavior at the household and community levels. The explanatory variables are also fundamentally different: this manuscript operationalizes bonding, bridging, and linking social capital as core predictors, while the governance study focuses on formal institutional arrangements, regulatory quality, and management structures.

Thus, despite being situated in the same regional context, the two papers address different scholarly debates – one contributing to the literature on social capital and sustainable rural development, and the other to institutional governance and common-pool resource management. The present article stands independently in its theoretical framing, empirical strategy, and contributions, without conceptual or analytical overlap.

## 2. Research area and methods

### 2.1. Research area

The research was conducted in Tan Son and Thanh Son, two mountainous districts located in the western part of Phu Tho province, in Vietnam’s northern midland and mountainous region. Both districts are situated in close proximity to Xuan Son National Park, one of the country’s recognized protected areas rich in biodiversity and cultural heritage.

Tan Son and Thanh Son are characterized by rugged topography, with a mix of low mountains, hills, and valleys. The climate is tropical monsoon with distinct wet and dry seasons, which supports both agriculture and forest ecosystems. Xuan Son National Park, which lies within the administrative boundaries of these districts, covers an area of over 15,000 hectares, consisting mainly of tropical evergreen forests, landscapes, and limestone caves. The park is home to a variety of rare plant and animal species, many of which are listed in Vietnam’s Red Data Book. The surrounding buffer zones of the park serve as living areas for various ethnic minority groups, notably the Muong, Dao, and Kinh. These communities rely heavily on the natural environment for their livelihoods, including farming, forest product collection, and increasingly, community-based tourism.

Both districts are part of Vietnam’s socio-economically disadvantaged areas, receiving targeted development support from government programs such as the National Target Program on Sustainable Poverty Reduction. Agriculture remains the dominant livelihood, with rice, maize, tea, and cassava being the primary crops. Livestock husbandry and small-scale forestry are also important, while non-timber forest products such as bamboo, medicinal herbs, and wild honey supplement household incomes. In recent years, there has been a gradual shift toward diversified livelihoods with the growth of ecotourism around Xuan Son National Park. Homestay tourism, cultural performances, and eco-trekking have provided alternative income sources, especially for younger and more dynamic households. However, access to markets, infrastructure, and training remains limited, particularly in remote villages.

Ethnic minority traditions play a significant role in local life, with strong social cohesion and customary practices governing resource use, conflict resolution, and community events. social organizations such as the Farmers’ Union, Women’s Union, and Youth Union are active in both districts, facilitating community mobilization and policy implementation.

Local governments in Tan Son and Thanh Son, in collaboration with Xuan Son National Park authorities and development partners, have implemented programs aimed at promoting sustainable livelihoods, forest protection, and community-based ecotourism. However, challenges remain in balancing conservation goals with economic development, particularly under conditions of poverty, limited education, and institutional capacity constraints.

### 2.2. Data collection and analysis

#### 2.2.1. Data collection.

This study adopted a mixed-method approach, integrating both quantitative and qualitative data collection and analysis to explore the role of social capital and dynamics in sustainable economic development and natural resource management in Xuan Son and Thanh Son districts, Phu Tho Province. While purely qualitative ethnographic studies can capture deep social dynamics, they lack generalizability. Conversely, quantitative methods alone may overlook context-specific nuances. The use of multiple sources and methodological triangulation enhanced the validity and reliability of the research findings.

Before initiating the fieldwork, verbal consent to conduct the survey was secured from the relevant local authorities, including district officials and the park’s management board. Only adult participants were invited, and their involvement was entirely voluntary. Prior to each interview, the objectives, procedures, and voluntary nature of the study were clearly explained to all participants. Verbal informed consent was obtained from each participant; this consent process was documented in the field notes and witnessed by at least one member of the research team. The use of documented verbal consent was reviewed and approved by the Departmental Ethics Review Committee. No personally identifiable details or sensitive data were recorded. The study involved minimal risk and complied with local administrative requirements, so formal institutional ethical approval was not necessary under Vietnamese regulations.

A stratified random sampling technique was employed to ensure representation across various ethnic groups, especially representation of major ethnic groups (Dao, Muong, Kinh), gender, household economic conditions, and levels of engagement in natural resource management. To ensure representativeness across ethnic groups, households were grouped by ethnicity, with samples proportionally allocated and randomly selected within each group. This ensured adequate representation of all major ethnic groups and reduced sampling bias. A total of 355 households were surveyed, selected from 6 communes within the districts based on their proximity to the national park. The sampling frame was derived from commune household lists.

Structured questionnaires were administered to 355 households. The survey captured quantitative data on:

- Levels of trust and social relationships within the community
- Information sharing practices (livelihood, environmental knowledge, market access)
- Participation in social organizations
- Involvement in ecotourism and environmental protection activities

The questionnaires were pre-tested for clarity and cultural appropriateness and were administered in the local language.

To complement survey data, 30 in-depth interviews were conducted with local leaders, tourism actors, and representatives from social organizations (e.g., Farmers’ Union, Women’s Union). Participants were selected through purposive sampling to ensure diversity in gender, age, livelihood strategies, and levels of engagement in forest management activities. All interviews were conducted on an individual basis. Data collection took place primarily in participants’ private residences, and no local authorities or external officials were present during the interviews in order to minimize potential influence or response bias.

Whenever possible, interviews were carried out in private settings to facilitate open and candid discussion. Prior to participation, respondents were informed about the purpose of the study and were assured that their identities would remain anonymous and that all information would be treated confidentially. Interview questions were carefully formulated using neutral and non-directive language to avoid leading responses. Topics that could be perceived as sensitive, particularly those related to institutional performance or compliance with regulations, were addressed indirectly and framed in a manner designed to reduce social desirability bias and perceived evaluative pressure.

A total of six focus group discussions were conducted to explore community-level perceptions of trust, cooperation, and collective action in tourism development and natural resource management. To facilitate open dialogue and reduce hierarchical or social constraints, groups were organized separately by gender and age. This segregation was intended to create a more comfortable environment for participants who might otherwise be reluctant to express their views in mixed or intergenerational settings.

Participants were selected using purposive sampling to ensure diversity in livelihood strategies, degrees of involvement in natural resource management, and socio-demographic characteristics. Each group was composed to reflect variation within these criteria while maintaining relative homogeneity in gender and age to encourage discussion.

All focus group discussions were moderated by the research team using a semi-structured guide. Moderators actively encouraged contributions from quieter participants and carefully managed group dynamics to prevent dominance by local elites or particularly vocal individuals. With the informed consent of participants, discussions were taken notes to ensure accuracy in transcription and subsequent analysis.

Relevant secondary sources were collected from local government reports, national park management documents, NGO project evaluations, and academic studies. These documents provided background on regional development strategies, and socio-economic statistics.

#### 2.2.2 Data analysis.

Quantitative data from the household surveys were entered and analyzed using SPSS software (version 22.0). Descriptive statistics, including frequencies, percentages, means, were used to summarize household characteristics, trust levels, and participation in social and economic activities. Cross-tabulation and correlation analyses were conducted to examine relationships between trust, social relationship and organizational participation.

Qualitative data from interviews and focus group discussions were transcribed and analyzed. Thematic analysis focused on patterns related to social capital, community cooperation, and challenges in sustainable resource management. These narratives helped to interpret and contextualize the quantitative findings rather than to serve as standalone confirmatory evidence. Its primary role was to illuminate mechanisms underlying statistical relationships and to strengthen the policy-relevant interpretation of results.

All key constructs were operationalized using five-point Likert scales ranging from 1 to 5. Although Likert-scale data are ordinal in nature, they were treated as continuous variables in the regression analysis. This approach is commonly adopted in social science research when Likert scales have five or more categories and approximate interval properties, as parametric methods have been shown to be robust under these conditions [30,31]. Treating these variables as continuous enables the use of Ordinary Least Squares (OLS) regression to estimate relationships between variables.

Composite indices were then created for each social capital dimension: Trust between households (TBH), social relationships (SR), participation in socio-political organizations (SO) and information sharing between households (IS). Internal consistency of these indices was tested using Cronbach’s Alpha, confirming reliability for quantitative analysis.

TBH was measured through the survey question: “How is the level of trust among households in daily life as well as in natural resource management?”. Responses were rated on a five-point Likert scale from 1 to 5: 1 – There is very little or no trust among households, 2 – Some trust exists, but households are still cautious or doubtful toward each other, 3 – A moderate level of trust; households trust each other in certain situations, 4 – Most households trust and cooperate with each other in daily and resource-related activities, 5 – Almost all households have strong mutual trust and willingly support each other.

SR was measured through the survey question: “How is the relationships among households in daily life as well as in natural resource management?”. Responses were rated on a five-point Likert scale from 1 to 5: 1 – Very few or no relationships exist; households rarely communicate, 2 – Some relationships exist but are weak or infrequent, 3 – Moderate relationships; households sometimes participate together in community activities, 4 – Households frequently interact and cooperate in various daily or management activities, 5 – Relationships are very close; households regularly coordinate and support each other.

SO was measured through the survey question: “How is the level of participation of household members in socio-political organizations (e.g., Farmers’ Union, Women’s Union, community forest management board)? Responses were rated on a five-point Likert scale from 1 to 5: 1 – Participating, but only formally, with almost no real involvement, 2 – One or two members participate, but their engagement is low and attendance is infrequent, 3 – A moderate level of participation; some members take part and occasionally contribute ideas or activities, 4 – Active participation; members are regularly involved and hold certain responsibilities, 5 – Very active participation; several members play key or leadership roles within organizations.

SI was measured through the survey question: “How is the sharing of information among the households in the community?”. Responses were rated on a five-point Likert scale from 1 to 5: 1 – There is very little or no sharing among households, 2 – Some sharing exists, but households are still cautious or doubtful toward each other, 3 – A moderate level of sharing; households share each other in certain situations, 4 – Most households share information and cooperate with each other in daily and resource-related activities, 5 – Almost all households have strong mutual information sharing.

Participating in the management and protection of forests and forest land (PMFL) was measured through the survey question: “How often does your household participate in forest protection and management activities (patrolling, tree planting, attending community meetings)?”. Responses were rated on a five-point Likert scale from 1 – never to 5 – very frequently. The variable was treated as continuous, consistent with prior studies applying OLS to Likert-based participation data.

**Fig 1:**
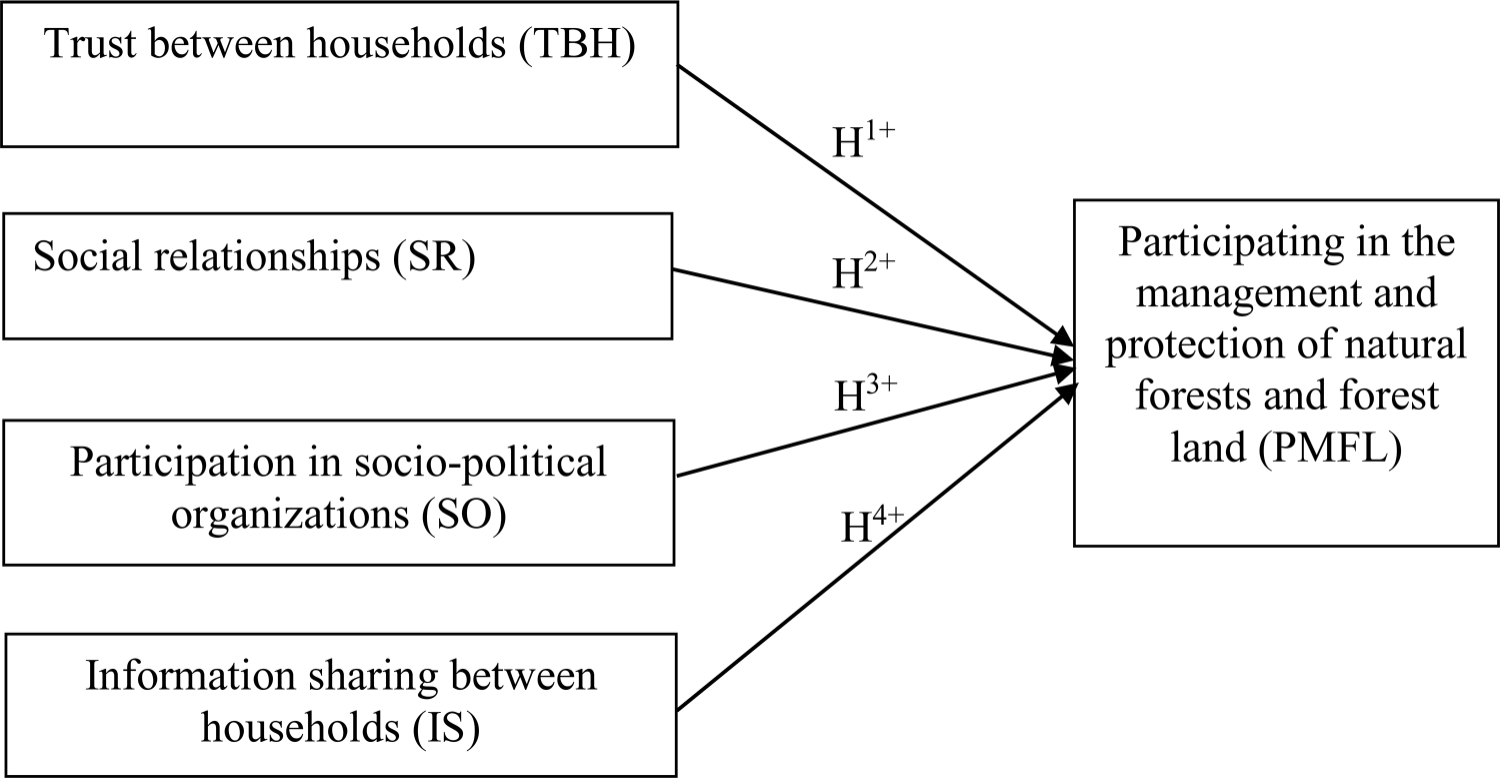
Model assessing the association between social capital and household participation in forest management and protection.

In order to indentify the association of social capital on participation in forest and forest land resource management activities of household, the reseach develops a linear regression model between them (Fig 1).

* Research Hypotheses

H0: The key factors have no correlation with participating in the management and protection of forests and forest land of households.

H1: Trust Between Households has a positive (+) impact on participating in the management and protection of forests and forest land of households.

H2: Social Relationship has a positive (+) impact on participating in the management and protection of forests and forest land of households.

H3: Participation in Socio-political organizations has a positive (+) impact on participating in the management and protection of forests and forest land of households.

H4: Information sharing between households has a positive (+) impact on participating in the management and protection of forests and forest land of households.

forest management and protection

* Regression model:

PMFL = β0 + β1* TBH + β2* SR + β3* SO + β4* IS + ε_i_

In which:

- PMFL: The dependent variable, representing household participation in the management and protection of natural forests and forest land.
- TBH: The first independent variable, capturing trust between households
- SR: The second independent variable, reflecting social relationships within the community
- SO: The third independent variable, indicating participation in socio-political organizations
- IS: The fourth independent variable, measuring information sharing between households
- β_0_: Constant
- β_i_: Coefficients of partial regression (i > 0)

ε_i_: Error term of the regression equation

The linear regression model was estimated using Ordinary Least Squares (OLS) in SPSS 22.0. OLS was chosen because Likert-scale variables with five or more categories are commonly treated as approximately continuous in social science research, allowing for reliable and interpretable estimation of relationships. This approach enables the assessment of the direct influence of independent variables on household engagement in forest management. In addition, OLS avoids the proportional odds assumption required by ordered logistic regression, making it a suitable and robust choice for this analysis. Prior to estimation, diagnostic tests confirmed no multicollinearity (VIF < 3.0) and normally distributed residuals, validating the model’s suitability.

## 3. Results and discussion

### 3.1. Trust and social relationships

**Fig 2:**
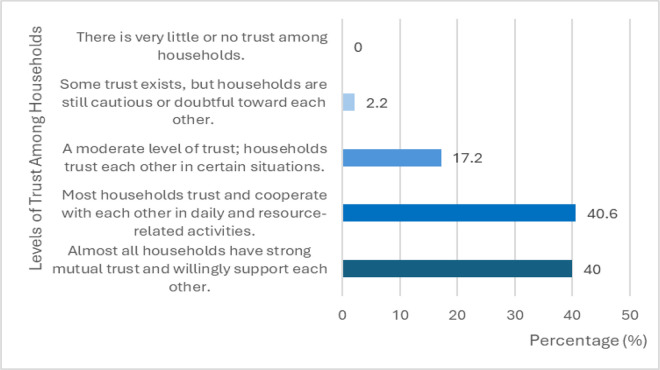
Distribution of reported trust levels (TBH) among surveyed households (N=355).

The research findings from Table 1 and Fig 2 provide data on the levels of trust and social relationships among households in the local ethnic minority community. The findings reveal that 80.6% of households reported either “Almost all households have strong mutual trust and willingly support each other” or “Most households trust and cooperate with each other in daily and resource-related activities” in their interactions with other households. Remarkably, only 2.2% of households indicated ”Some trust exists, but households are still cautious or doubtful toward each other”. This extremely high level of mutual trust reflects a strong foundation of social capital within the community. Such trust is a key prerequisite for cooperative behavior, reducing transaction costs, and enabling shared responsibilities in local economic development initiatives.

**Table 1:** Descriptive statistics for trust and social relationship variables (N = 355).

No.	Criteria	Level	Frequency (households)	Percentage (%)
1	Trust	Almost all households have strong mutual trust and willingly support each other.	142	40.00
		Most households trust and cooperate with each other in daily and resource-related activities.	144	40.60
		A moderate level of trust; households trust each other in certain situations.	61	17.20
		Some trust exists, but households are still cautious or doubtful toward each other.	8	2.20
		There is very little or no trust among households.	0	0.00
2	Social relationship	Relationships are very close; households regularly coordinate and support each other.	112	31.50
		Households frequently interact and cooperate in various daily or management activities.	140	39.50
		Moderate relationships; households sometimes participate together in community activities.	96	27.00
		Some relationships exist but are weak or infrequent.	6	1.70
		Very few or no relationships exist; households rarely communicate.	1	0.30

Trust between households (TBH) was measured on a five-point Likert scale (1 = very low trust; 5 = very high trust). Values represent the percentage of households in each category.

**Fig 3:**
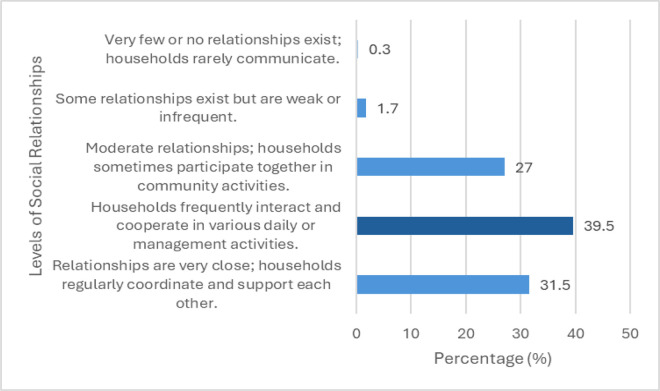
Distribution of social relationship levels (SR) among surveyed households (N=355).

In parallel, interpersonal relationships are also notably strong. Findings from Table 1 and Fig 3, 71% of households reported that their relationships with others were either “Households frequently interact and cooperate in various daily or management activities” or “Relationships are very close; households regularly coordinate and support each other” with just two percent of households reporting “Some relationships exist but are weak or infrequent” or ”Very few or no relationships exist; households rarely communicate”. The results indicate a high degree of community cohesion, which is statistically linked to building local networks for collaborative economic activities. In the context of ecotourism and sustainable livelihoods, such strong social ties enable households to form working groups, co-manage community-based tourism services, and jointly protect natural resources.

Social relationship (SR) were assessed on a five-point Likert scale ranging from 1 (very limited ties) to 5 (very strong, cooperative ties). Reported values indicate the percentage of households in each category.

Trust and close social relations further enhance the effective sharing of information within the community. Information related to livelihood strategies, market demands, environmental conservation, and cultural practices could be shared freely and rapidly, enhancing households’ capacity to make informed decisions [4,13]. These dynamics play a crucial role in risk reduction and knowledge diffusion, particularly in rural and vulnerable areas such as the buffer zones of national parks, where communities often face economic uncertainties [32].

Moreover, strong mutual trust and social bonds facilitate value chain development beyond individual households or kinship groups. Households are more willing to engage in joint ventures, share labor and equipment, and participate in collective decision-making processes [4,13]. This created an environment conducive to sustainable economic development, where growth is inclusive, community-led, and environmentally responsible. It also supported the emergence of cooperative models such as producer groups, community-based tourism cooperatives, and environmental stewardship initiatives [32].

Results from Table 1 highlight that social cohesion and trust were not only social assets but also economic enablers. They empower local communities to collectively manage resources, develop sustainable livelihoods, and build resilience to external shocks. Therefore, fostering and maintaining strong interpersonal trust and social connections should be a core component of sustainable development strategies in rural and ethnic minority areas.

The research findings align with existing research emphasizing the role of trust and social cohesion in fostering sustainable economic development and resource management in rural and ethnic minority communities. For example, Pretty and Ward (2001) argued that social capital, particularly mutual trust and shared norms, acts as a catalyst for collective action, enabling rural communities to manage common-pool resources more sustainably [13]. In contexts where formal institutional support is limited, communities with high levels of interpersonal trust are more likely to coordinate the use of forest, water, land, and biodiversity resources in ways that reduce overexploitation and conflict. This reflects the situation in Tan Son, where mutual trust among households provides a stable social foundation for community-based tourism and participatory environmental stewardship.

Similarly, Narayan and Pritchett (1999), in their seminal study on social capital in Tanzanian villages, find that stronger trust and cooperation correlated with better access to shared infrastructure, services, and economic opportunities. They noted that strong internal networks reduce the costs of collective action, making it easier to maintain irrigation systems, protect forests, and develop community-based enterprises, key aspects of sustainable resource governance [33]. The findings from Tan Son reflected a similar pattern, where strong horizontal ties are likely to support joint forest patrols, community monitoring of ecotourism impacts, and equitable resource sharing.

Woolcock and Narayan (2000) also emphasize the value of “embedded” social relationships for managing environmental and economic uncertainty. In their view, dense local networks promote information flow, mutual monitoring, and peer enforcement, all of which are essential in the management of shared natural assets [4]. In the context of Tan Son, the high levels of trust and social closeness reported in Table 1 could facilitate the creation of informal rules and norms around ecotourism zoning, waste management, or rotational use of natural sites, which are core elements of community-based natural resource management.

In ecotourism settings, Stronza and Gordillo (2008) demonstrate that communities with cohesive social structures are better positioned to establish rules for visitor access, reinvest tourism revenue into conservation, and manage visitor behavior in culturally and ecologically sensitive areas. Their research in Latin America find that internal trust was directly linked to long-term conservation outcomes because it reduced elite capture and increased accountability within tourism cooperatives [32]. This resonates with the Tan Son case, where high trust and strong social ties are likely to foster shared stewardship of forest landscapes, enabling locals to protect biodiversity while benefiting economically.

In the Vietnamese context, ethnic minority communities in the Central Highlands who rely on traditional institutions and trust-based relationships are more effective in managing forest resources and adapting to land use policy changes. These informal social systems often function alongside, or in place of, state-led conservation programs. In northern Vietnam, trust among villagers led to the creation of self-managed environmental groups and collaborative livelihood models, such as forest protection teams or agroforestry collectives. These examples support the idea that social trust serves as an informal but powerful governance mechanism, reducing reliance on external enforcement and fostering sustainable practices from within.

By contrast, in communities where social capital is weak or fragmented, efforts to manage resources collectively often fail due to distrust, free-riding, and conflict. These communities are more dependent on outside interventions, which are often costly, unsustainable, or culturally incompatible. The presence of trust and the prevalence of close interpersonal bonds in Tan Son therefore represent more than just favorable social conditions – they constitute a strategic asset for sustainable resource management and local resilience.

### 3.2. Information sharing

The study results indicate that information-sharing channels within the community are diverse and highly active (Table 2). Information is frequently exchanged among family members, relatives, members of the same ethnic group, villagers, and even those working in similar occupations. The sharing rates across most channels are notably high, except among households within the same occupational group, likely reflecting the underdevelopment of traditional occupations in the area. This reflects a strong sense of connection and communication among community members.

**Table 2:** Descriptive characteristics of information sharing (N = 355).

**No.**	**Level of sharing**	**Frequency (No. of households)**	**Percentage (%)**
1	With family members	345	97.18
2	With relatives	305	85.92
3	With people of the same ethnic group	283	79.72
4	With households in the same occupation group	52	14.65
5	With people in the same village/commune	236	66.48

**Fig 4:**
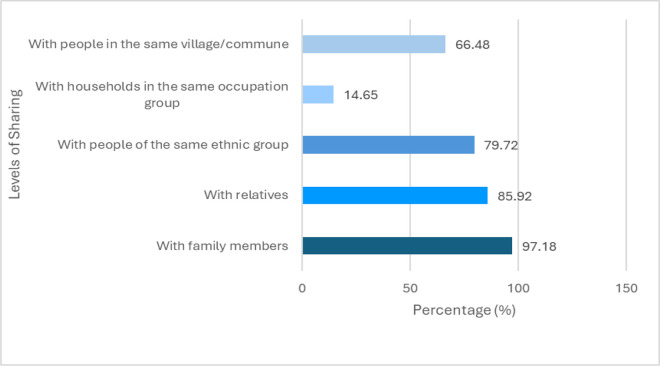
Distribution of information sharing levels among community members (N=355).

The research results reveal that information sharing is most prevalent within intimate social networks (Fig 4), particularly among family members (97.18%), relatives (85.92%), and individuals of the same ethnic group (79.72%). This pattern reflects a high degree of bonding social capital.

Information sharing (IS) was measured based on reported information exchange across social groups. Values indicate the percentage of households reporting information sharing with each group.

These findings align with Woolcock and Narayan (2000) and Pretty and Ward (2001), who argue that close social ties support the rapid dissemination of context-specific knowledge, which is crucial for adaptive livelihoods and environmental stewardship in marginalized rural areas [4,13]. In Vietnamese, Tran and Walter (2014) find that in ethnic minority villages in the Central Highlands, kinship and ethnic ties were the primary pathways for information sharing about farming techniques and forest conservation practices [34].

However, the low frequency of information sharing with peers within the same occupation (14.65%) or even other villagers (66.48%) suggests limitations in bridging social capital. This inward-looking dynamic can pose challenges for scaling up community-based development, especially for ecotourism and natural resource co-management, which require coordination across households, market knowledge, and technical innovation [35].

Where community-based tourism has been successful, such as in Laos, Nepal, and parts of Vietnam, structured platforms such as cooperatives, producer groups, and training networks have been key to expanding communication channels beyond ethnic and family ties [36]. These platforms foster trust among previously disconnected groups and integrate external actors, including NGOs and government agencies, into community planning.

Furthermore, information sharing that extends beyond kin groups plays a vital role in sustainable resource management. Olsson, Folke, and Hahn (2004) show that successful community forest governance often involves network leaders who act as knowledge brokers, connecting local knowledge holders with scientific and policy communities [37].

Thus, while the results demonstrate a strong cultural foundation for information sharing, they also highlight the need to broaden these networks through institutional mechanisms that promote bridging capital. This can help communities in buffer zones enhance their economic resilience, adaptive capacity, and collective action potential in the face of market volatility and environmental change.

### 3.3. Participation in community organizations

The level of participation in associations, unions, and community groups reflects an important aspect of each member’s social capital. Participation in these organizations provides practical benefits for socio-economic development, including access to credit, access to information on government policies and legal regulations, as well as opportunities for information sharing.

**Fig 5:**
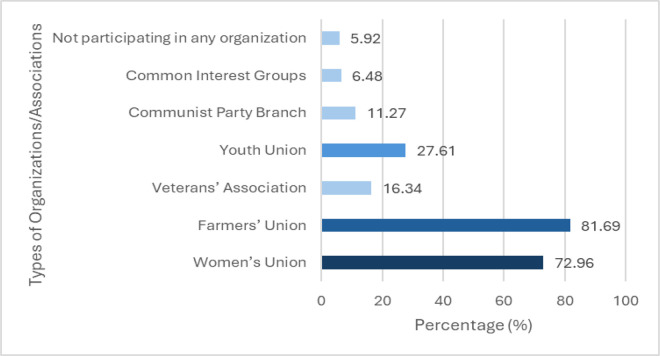
Distribution of household participation in community organizations (N=355).

Findings from Table 3 and Fig 5 reveal that community engagement in formal organizations is relatively high in certain domains (agriculture and women’s affairs organizations), but remains low in market-oriented, collaborative, and political structures. Enhancing participation in interest-based and cross-sectoral organizations could improve capacity for sustainable economic development and resource governance, especially in ecotourism, conservation, and climate adaptation initiatives.

**Table 3:** Descriptive statistics on household participation in community organizations (N = 355).

No.	Type of organization/association	Frequency (households)	Percentage (%)
1	Women’s Union	259	72.96
2	Farmers’ Union	290	81.69
3	Veterans’ Association	58	16.34
4	Youth Union	98	27.61
5	Communist Party Branch	40	11.27
6	Common Interest Groups	23	6.48
7	Not participating in any organization	21	5.92

Table 3 and Fig 5 provide insights into the organizational engagement of households in the study area, with the Farmers’ Union (81.69%) and the Women’s Union (72.96%) showing the highest levels of participation. Other organizations such as the Youth Union (27.61%), Veterans’ Association (16.34%), and Communist Party Branch (11.27%) show lower levels of participation, while Common Interest Groups (6.48%) and the number of households not participating in any organization (5.92%) remain relatively low.

Participation in socio-political organizations (SO) was measured as membership status across the listed organizations. Values represent the percentage of households participation in each organization.

This distribution indicates a strong presence of formal mass organizations, particularly those traditionally supported by the Vietnamese state, within rural and ethnic minority communities. These organizations play a critical role in social mobilization, knowledge dissemination, and implementation of development programs, including those related to livelihood improvement, gender equality, and environmental conservation [38].

The high participation in the Farmers’ Union and Women’s Union aligns with findings by Beckman (2001), who observed that these organizations often serve as primary conduits for agricultural extension, credit access, and environmental awareness among ethnic minorities. They help facilitate information flows and trust building, especially in contexts where informal networks dominate and state outreach is limited [39].

The Women’s Union, in particular, is instrumental in promoting sustainable resource use, such as fuel-saving stoves and clean water initiatives [34]. Its leadership often overlaps with community-based tourism and forest co-management efforts in upland and buffer zone areas.

Low participation in common interest groups (6.48%) is notable. These groups, typically established to connect producers with markets, cooperatives, or ecotourism initiatives, play a crucial role in value chain development and scaling of sustainable livelihoods [36]. Their weak presence may reflect a lack of institutional support, training, or incentives for collective economic action.

According to Ostrom (1990) and Berkes (2009), strong collective organizations are crucial for managing common resources effectively. Without broader participation in such groups, communities may struggle to coordinate environmental protection or distribute tourism benefits equitably [10,35].

Participation in the Communist Party Branch (11.27%) is low, which may suggest limited political representation or engagement in decision-making processes. This can be a barrier to inclusive governance, especially in areas where resource allocation and conservation policies are determined by state actors or external NGOs.

The combination of formal participation in mass organizations and informal trust networks illustrates a dual-layered social capital system. As Pretty and Ward (2001) argue, this combination of bonding and bridging capital is essential for community, driven sustainable development, where trust facilitates cooperation, and institutions help scale these processes [13].

Communities with strong participation in organizations tend to be better equipped to respond to economic shocks, climate risks, and environmental challenges, as collective action becomes more feasible and information becomes more accessible [33].

### 3.4. Building a linear regression model between social capital and household participation in forest and forest land resource management activities

The regression model in Table 4 reveals valuable insights into the relationship between components of social capital, namely trust between households (TBH), social relationships (SR), participation in social organizations (SO), and information sharing between households (IS) – and a dependent variable associated with household engagement in sustainable resource management.

**Table 4:** Summary of multivariate regression model.

Model summary				
Model	R	R square	Adjusted R square	Std. error of the estimate
1	0.349^a^	0.122	0.112	0.6638

a. Predictors: (Constant), TBH (trust between households), SR (social relationships), SO (participation in social organizations), IS (information sharing between households)

b. Dependent variable: PMFL (Participating in the management and protection of natural forests and forest land).

The multiple correlation coefficient (R) of 0.349 indicates a moderate positive association between the independent variables and the outcome, implying that increases in trust, social connectedness, and organizational involvement are associated with higher levels of household participation in the target activity. The coefficient of determination (R²) is 0.122, which means that approximately 12.2% of the variance in the dependent variable can be attributed to the combined association of TBH, SR, SO and IS. While this value may appear modest, it is consistent with expectations in social research, where behavior is often associated by a wide range of contextual and structural factors. The adjusted R², which accounts for the number of predictors and the sample size, slightly reduces the explained variance to 11.2%, reinforcing the reliability of the model without overfitting the data. This moderate value indicates a reasonable fit, but also highlights room for improving the model’s predictive power by incorporating additional explanatory variables, such as household income, education level, land tenure status, or external institutional support. Overall, the model demonstrates that elements of social capital play an important role in shaping household-level engagement, but should be considered alongside other economic and institutional drivers to more fully understand sustainable behavior in resource-dependent communities.

The ANOVA (Analysis of Variance) results in Table 5 provide a statistical assessment of the overall significance of the regression model, which examines the association between social capital variables, including TBH, SR, SO, and IS on household involvement in PMFL. The regression sum of squares is 21.370 with four degrees of freedom, indicating that a portion of the variation in PMFL is explained by these four predictors. In contrast, the residual sum of squares stands at 154.219 with 350 degrees of freedom, representing the portion of variation not captured by the model.

**Table 5:** Overall fit statistics for the linear regression model.

ANOVA^a^						
Model		Sum of squares	Df	Mean square	F	Sig.
1	Regression	21.370	4	5.342	12.125	0.000^b^
	Residual	154.219	350	0.441		
	Total	175.589	354			

a. Dependent Variable: PMFL (Participating in the management and protection of natural forests and forest land)

b. Predictors: (Constant), TBH (trust between households), SR (social relationships), SO (participation in social-political organizations), IS (information sharing between households).

The model’s total sum of squares is 175.589, representing the total variability in the dependent variable. The F-statistic value of 12.125 reflects the ratio of the variance explained by the model to the unexplained variance. This relatively high F-value indicates that the regression model performs significantly better than a null model in accounting for differences in PMFL.

Most notably, the associated p-value (p < 0.001) is well below the standard 0.05 threshold, confirming that the model is statistically significant overall. This means that the combined effects of TBH, SR, SO and IS contribute to explaining household-level engagement in natural resource management. The findings underscore the importance of social capital as a driving factor in promoting sustainable practices at the community level, and suggest that efforts to enhance interpersonal trust, strengthen social ties, expand participation in community organizations, and improve information sharing may have a direct and measurable impact on forest governance and environmental stewardship.

The regression results in Table 6 provide detailed insights into the individual associations of four components of social capital – TBH, SR, SO and IS – with household participation in PMFL. Each of these variables shows a statistically relationship with the dependent variable, indicating that they each contribute to explaining variations in PMFL.

**Table 6:** Results of the multiple regression analysis.

Coefficients^a^								
Model		Unstandardized coefficients		Standardized coefficients	T	Sig.	Collinearity statistics	
		B	Std. error	Beta			Tolerance	VIF
1	(Constant)	1.591	0.250		6.371	0.000		
	TBH	0.146	0.069	0.139	2.113	0.035	0.578	1.729
	SR	0.132	0.062	0.143	2.145	0.033	0.567	1.762
	SO	0.78	0.32	0.127	2.445	0.015	0.933	1.072
	IS	0.066	0.027	0.127	2.400	0.015	0.928	1.077

a. Dependent variable: PMFL (Participating in the management and protection of natural forests and forest land).

The constant value (B = 1.591, p < 0.001) represents the expected level of PMFL when all predictors are zero. This base level is slightly above the lowest category of the scale and further confirms that this baseline value is statistically different from zero.

**Fig 6:**
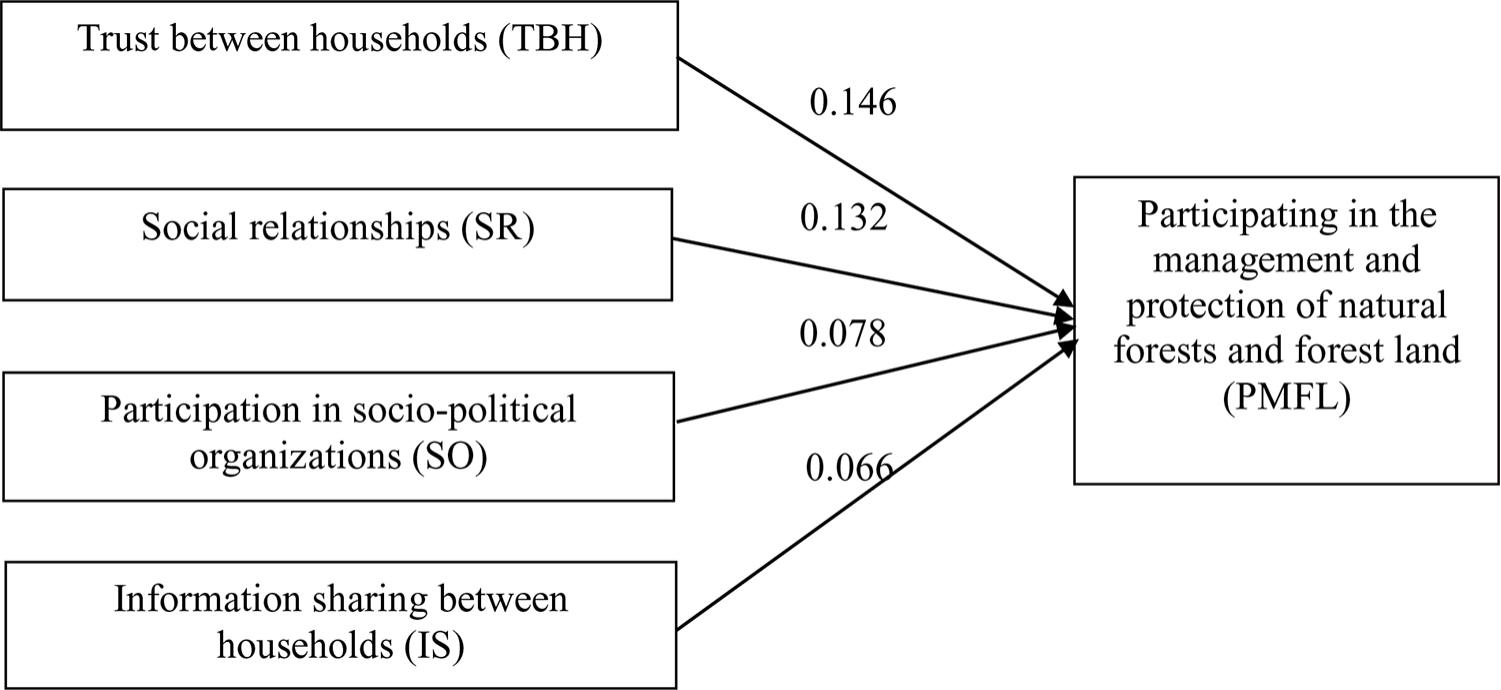
Regression framework linking social capital and household participation in forest management and protection.

The coefficient analysis (Table 6, Fig 6) reveals that all four predictors have positive and statistically significant influences on PMFL. Specifically, TBH shows a positive effect (B = 0.146, β = 0.139, p = 0.035), indicating that higher TBH are associated with higher PMFL. Similarly, SR also demonstrates a positive and significant impact (B = 0.132, β = 0.143, p = 0.033). Both SO (B = 0.078, β = 0.127, p = 0.015) and IS (B = 0.066, β = 0.127, p = 0.015) also contribute positively to the prediction of PMFL, although their standardized effects are comparatively smaller.

Regression model:

PMFL = 1.591 + 0.146 * TBH + 0.132 * SR + 0.078 * SO + 0.066 * IS + ε_i_

management and protection

Multicollinearity diagnostics indicate no concerns, with tolerance values ranging from 0.567 to 0.933 and VIF values between 1.072 and 1.762, all well within acceptable thresholds (VIF < 3.0). This confirms that the predictors do not substantially overlap in their explanatory contributions and are statistically suitable for inclusion in the same regression model.

Together, these results confirm that trust, social ties, organizational involvement and information sharing of social capital are drivers in enhancing community-based natural resource governance. Strengthening these aspects can therefore be a vital strategy in promoting sustainable and inclusive environmental management in rural and buffer zone areas.

Social capital within local communities represents a significant strength, characterized by high levels of trust among households, diverse channels of information sharing, and active participation in social organizations and information sharing between households. Such trust among community members creates favorable conditions for disseminating of government policies and new technologies. However, while trust is a positive attribute, it can also pose challenges to the adoption of new policies and scientific advancements. If respected individuals within the community or extended families are resistant to change, this high level of trust may inadvertently hinder progress by reinforcing traditional practices and reducing openness to innovation.

Overall, these findings suggest that improvements in TBH, SR, SO, and IS are each associated with higher levels of PMFL. This study contributes to the literature by demonstrating that social capital is not a single construct but a set of interrelated dimensions that collectively shape household participation in forest and land management. Rather than examining these elements in isolation, the analysis integrates trust, social relationships, organizational participation, and information sharing within a single empirical framework. The results indicate that both close-knit community ties and broader forms of engagement beyond the household context play important roles in explaining participation outcomes. This provides stronger empirical support for the argument that social capital influences environmental behavior through multiple pathways at the local level.

In the context of community-based forest management, the findings offer insights into how collective action is sustained in practice. Strong interpersonal trust and frequent interaction among households appear to reduce coordination challenges, while participation in local organizations and active information exchange strengthen linkages with broader institutional structures. This suggests that effective forest governance depends not only on formal regulations but also on the underlying social relationships that facilitate cooperation and shared responsibility.

The study also contributes to the literature on ethnic minority development by providing quantitative evidence from upland Vietnam. The findings highlight a critical balance: strong internal cohesion supports participation in local resource management, whereas limited connections to external actors may constrain access to new knowledge, markets, and innovation. By combining statistical analysis with contextual insights, the study offers a more nuanced understanding of how social capital shapes development outcomes in resource-dependent communities.

In Tan Son and Thanh Son districts, where ethnic minority communities live in close proximity to Xuan Son National Park, natural resource management and sustainable development are deeply intertwined with social relationships and traditional practices. The findings of this study indicate that strong social capital exists in the form of high levels of trust, close family and clan ties, and active participation in local associations. To leverage these strengths for sustainable outcomes, the following strategies are recommended:

Strengthening community-based resource management institutions: Given the high degree of internal cohesion and trust among households, there is a strong foundation for developing or revitalizing community-based natural resource management models. Village-level forest protection teams, agricultural self-help groups, and water user associations should be established or reinforced with clear rules, inclusive participation, and transparent benefit-sharing mechanisms. These institutions can formalize customary practices and transform informal social ties into effective governance structures for forest, land, and water resources.

Capacity building and livelihood diversification: Training programs should be tailored to the specific needs of ethnic groups in Tan Son and Thanh Son, focusing on sustainable farming techniques, non-timber forest product harvesting, community forestry, and climate-resilient practices. Additionally, promoting diversified livelihoods, such as eco-friendly handicrafts, traditional herbal medicine, and community-based tourism, can help reduce pressure on natural resources while increasing household income. Community members, especially women and youth, should be the focus of such capacity-building initiatives to ensure intergenerational sustainability and equity.

Bridging traditional and modern knowledge: In Tan Son and Thanh Son, the authority of elders and respected figures in the community plays a key role in shaping local norms and attitudes. Development strategies should respect and integrate indigenous knowledge systems in conservation and land use planning. At the same time, efforts should be made to engage these influential figures in dialogue about innovation and change, particularly in response to climate variability and market shifts. This dual approach helps ensure that cultural identity is preserved while facilitating the adoption of sustainable practices.

Strengthening bridging and linking social capital: While bonding social capital in Tan Son and Thanh Son is strong, the study shows weaker ties with professional networks and cross-sectoral partnerships. Strategies should therefore focus on building connections with external stakeholders, including NGOs, research institutions, and private enterprises. This can be achieved through technical support projects, the development of cooperatives, and knowledge exchange programs. For example, linkages with agricultural universities or forestry research centers could provide scientific insights and innovations to complement local practices.

Participatory monitoring and evaluation: Empowering local communities in Tan Son and Thanh Son to develop participatory monitoring systems allows them to monitor the condition of their natural resources and the outcomes of development interventions. These systems can draw on both scientific indicators and community-defined success metrics. Regular reflection meetings, combined with visual tools such as resource maps or seasonal calendars, can promote learning and accountability while reinforcing collective responsibility.

Enhancing environmental education and awareness: To ensure the long-term sustainability of natural resource management, environmental education should be expanded in both formal and informal settings. Schools, cultural houses, and commune-level meetings can be used to raise awareness about biodiversity conservation, climate change adaptation, and sustainable livelihoods. Targeting youth and women in these efforts is crucial, as they are both highly engaged in community life and often act as change agents in household and village-level decision-making.

## 4. Conclusions

This study underscores the pivotal role of social capital in fostering sustainable economic development and natural resource governance among ethnic minority communities in Tan Son and Thanh Son districts, Phu Tho province, Vietnam. Through a comprehensive mixed-methods approach, the findings reveal that trust-based social relations, interpersonal networks, and organizational participation collectively form a robust framework for enabling community resilience, collaborative governance, and livelihood diversification, particularly in buffer zones adjacent to protected areas like Xuan Son National Park.

High levels of mutual trust (over 80%) and close interpersonal relationships (71%) serve as a foundation for collective efforts in forest management, ecotourism, and risk mitigation. Widespread information sharing within families and ethnic groups facilitates the diffusion of local knowledge, though limited bridging ties constrain broader innovation and inter-group collaboration. Meanwhile, high engagement in mass organizations – particularly the Farmers’ Union and Women’s Union – is instrumental in disseminating technical information, mobilizing community efforts, and supporting participatory development.

The regression analysis further confirms that social capital components, including trust between households, social relationships, and participation in socio-political organizations are all statistically significant predictors of household engagement in forest and forestland management. While these factors explain a modest share of the variance, their significance affirms that social capital is not merely a cultural asset but a strategic resource for sustainable development.

Importantly, the study identifies both opportunities and limitations. While bonding capital is positively related to internal cohesion, the lack of bridging and linking capital may hinder communities from accessing external markets, knowledge, and policy frameworks. This duality necessitates development strategies that reinforce existing social strengths while systematically expanding cross-sectoral networks and institutional capacities.

Investment in social capital should be central to policies targeting environmental sustainability and inclusive development in upland ethnic regions. Strengthening participatory institutions, fostering multi-stakeholder linkages, and promoting culturally embedded yet adaptive practices will be critical to realizing long-term socio-ecological resilience in Vietnam’s mountainous buffer zones.

Although the findings provide meaningful evidence that social capital, particularly trust, close social relations, and organizational participation, is strongly associated with household engagement in forest and forest land management, this study has some limitations. The cross-sectional design limits the analysis to associations rather than causal relationships between social capital and household participation in forest and forest land management. The model explains a moderate share of variance, indicating that other relevant factors, such as income, education, land tenure, and institutional support, were not fully included.

Future research should address these limitations by employing longitudinal data to better examine causal relationships and dynamic changes in social capital over time. Expanding the model to include additional socio-economic and institutional variables would improve explanatory power. Moreover, future research may apply alternative analytical approaches, such as structural equation modeling or multilevel analysis, to better capture the complex interactions between social capital, governance, and sustainable resource management.
